# Factors contributing to food insecurity among women living with HIV in the Dominican Republic: A qualitative study

**DOI:** 10.1371/journal.pone.0181568

**Published:** 2017-07-25

**Authors:** Kathryn P. Derose, Denise D. Payán, María Altagracia Fulcar, Sergio Terrero, Ramón Acevedo, Hugo Farías, Kartika Palar

**Affiliations:** 1 Department of Behavioral and Policy Sciences, RAND Corporation, Santa Monica, California, United States of America; 2 Department of Health Policy and Management, Fielding School of Public Health, University of California, Los Angeles, California, United States of America; 3 United Nations World Food Programme, Dominican Republic Country Office, Santo Domingo, Dominican Republic; 4 Consejo Nacional de VIH/SIDA (CONAVIHSIDA), Santo Domingo, Dominican Republic; 5 United Nations World Food Programme - Regional Bureau for Latin American and the Caribbean, Panamá, Rep. de Panama; 6 Division of HIV, ID and Global Medicine, School of Medicine, University of California - San Francisco, San Francisco, California, United States of America; Western Sydney University, AUSTRALIA

## Abstract

**Background:**

Food insecurity contributes to poor health outcomes among people living with HIV. In Latin America and the Caribbean, structural factors such as poverty, stigma, and inequality disproportionately affect women and may fuel both the HIV epidemic and food insecurity.

**Methods:**

We examined factors contributing to food insecurity among women living with HIV (WLHIV) in the Dominican Republic (DR). Data collection included in-depth, semi-structured interviews in 2013 with 30 WLHIV with indications of food insecurity who resided in urban or peri-urban areas and were recruited from local HIV clinics. In-person interviews were conducted in Spanish. Transcripts were coded using content analysis methods and an inductive approach to identify principal and emergent themes.

**Results:**

Respondents identified economic instability as the primary driver of food insecurity, precipitated by enacted stigma in the labor and social domains. Women described experiences of HIV-related labor discrimination in formal and informal sectors. Women commonly reported illegal HIV testing by employers, and subsequent dismissal if HIV-positive, especially in tourism and free trade zones. Enacted stigma in the social domain manifested as gossip and rejection by family, friends, and neighbors and physical, verbal, and sexual abuse by intimate partners, distancing women from sources of economic and food support. These experiences with discrimination and abuse contributed to internalized stigma among respondents who, as a result, were fearful and hesitant to disclose their HIV status; some participants reported leaving spouses and/or families, resulting in further isolation from economic resources, food and other support. A minority of participants described social support by friends, spouses, families and support groups, which helped to ameliorate food insecurity and emotional distress.

**Conclusions:**

Addressing food insecurity among WLHIV requires policy and programmatic interventions to enforce existing laws designed to protect the rights of people living with HIV, reduce HIV-related stigma, and improve gender equality.

## Introduction

The Caribbean region’s HIV prevalence rate is the second highest in the world after sub-Saharan Africa. HIV and food insecurity pose interrelated problems in Latin America and the Caribbean (LAC), as well as in other resource limited settings. People experiencing food insecurity—defined as limited or uncertain availability of nutritionally adequate, safe foods or the inability to acquire acceptable foods in socially acceptable ways [1, 2]—are more vulnerable to HIV infection due to their reduced immunity [3]. Food insecurity is also associated with HIV risk behaviors and factors, including drug use, risky sex, and having a sexually transmitted infection, even after accounting for other measures of socioeconomic status [4–7]. Among people living with HIV (PLHIV), food insecurity is of significant concern due to its harmful effects on health outcomes [8–13], independent of economic status, and has been found to be associated with reduced antiretroviral therapy (ART) adherence [14–19], reduced viral suppression [20, 21], and increased morbidity and mortality [22–26]. In addition, women and female-headed households have higher rates of food insecurity, and often occupy social roles (e.g. mothers, caretakers) that may intensify the negative impacts of food insecurity on mental and physical health [27, 28].

In LAC, structural factors such as poverty, stigma, and inequality drive the HIV epidemic [29] and may also be closely related to food insecurity; yet, the interplay between food insecurity and social inequality among PLHIV in the region has gone largely unexplored. Since social drivers, such as stigma, can be manifested in a variety of ways contingent on a specific sociocultural context [30–32], it is important to examine how these drivers are operationalized in specific locations and how they contribute to food insecurity among specific groups. Definitions and conceptualizations of food insecurity need to take into account social, cultural, and political contexts [33]. Further, more research is needed to explore the determinants of food insecurity among women living with HIV (WLHIV) in various cultural contexts, since gender inequities in intra-household resource allocation often mean that women bear more of the burden of food insecurity within households [2, 19, 34, 35]. Gender inequality feeds into and exacerbates economic inequalities including poverty, labor discrimination, and limited educational opportunities, and may make women more vulnerable to social factors including HIV-related stigma, lack of social support, and familial roles and expectations. Stigma manifests across settings and can result in decreased economic prospects and social isolation [32, 36, 37] and potentially perpetuate food insecurity through multiple pathways.

A conceptual framework by Weiser et al. [22] identifies three levels of determinants influencing food insecurity and HIV linkages: community, household, and individual. A nuanced relationship exists among these determinants. For instance, community-level structural drivers, such as ecologic (e.g., drought, floods), economic (e.g., poverty, education), and social factors (e.g., gender, stigma), can influence food insecurity at the household level which, in turn, can lead to worse health outcomes at the individual level for PLHIV and people at risk for HIV through three main pathways: nutritional (micronutrient deficiencies, undesired weight loss or gain), mental health (anxiety, depression, substance use), and behavioral (poor engagement in health care). However, the linkages are complex since these relationships can work in multiple directions. For example, HIV can increase food insecurity through economic pathways, such as decreased productivity among key household wage earners [38] and loss of assets due to unexpected medical expenditures or absenteeism [3, 34, 39, 40], and it can exacerbate structural vulnerabilities like stigma and lack of social support, which can further distance individuals from food support systems [22, 40]. It is also important to note the bi-directional nature of the relationship between food insecurity and HIV, whereby food insecurity makes people more vulnerable to HIV infection and the hastening of disease progression [41]. Further work is needed to explicate factors affecting PLHIV’s ability to access food resources in LAC regions, since, to date, most of the evidence regarding the relationship among poverty, food insecurity, and HIV has been conducted in Sub-Saharan African countries and among vulnerable populations in the United States (U.S.) [22, 34, 40, 42].

In this paper, we explore how economic and social structural factors contribute to food insecurity among WLHIV in the Dominican Republic (DR) using in-depth, semi-structured interviews conducted in Spanish. We use Weiser et al.’s multi-level framework [22] to conceptualize the linkages between food insecurity and HIV and focus our attention on the antecedent, structural drivers of food insecurity with an emphasis on economic and social factors rather than on the subsequent influence of food insecurity on the risk of HIV acquisition, transmission, and health-related outcomes. A qualitative study that seeks to explicate the pathways by which economic and social factors contribute to food insecurity among WLHIV can provide important contextual data that is needed to inform and potentially shift programmatic interventions and policy responses to address food insecurity and improve the health and wellbeing of this vulnerable population.

## Methods

### Study context

The DR has the highest general HIV prevalence among Spanish-speaking countries in LAC. In 2014, HIV prevalence in the DR was estimated to be 1.0% among individuals aged 15 to 49 years old or approximately 69,000 people infected [43]. Similar to other Caribbean countries, reported HIV transmission in the DR is predominantly due to unprotected heterosexual sex and the infection rate has been disproportionately increasing among women [44]. Over half of adults living with HIV in the DR and the Caribbean are women [45], unlike most other Latin American countries where HIV rates are higher among men [46].

The DR has an estimated population of 10.4 million people, with a majority of residents residing in urban areas (78%) [47, 48]. Although the World Bank classifies the country as upper middle income, inequalities are widespread and over 40% of the country’s population lives below the national poverty line [49]. Women are particularly vulnerable to economic inequality and encounter gender inequality in the economic and social domains. Women have higher unemployment rates compared to men, and employed women only receive about 0.44 cents for every dollar earned by men in the DR [50]. The 2008 global economic crisis further exacerbated economic inequalities, as it led to declines in tourism—an industry that comprises an important part of the country’s economy (15–20% of the country’s GDP) and employs a large number of women [51]. Between 2004 and 2013, employment in the informal sector increased in the country; this type of work often has fewer legal protections and benefits for workers [52]. In terms of gender equality, domestic violence is a persistent issue. About a third of Dominican women report being physically abused by their spouse/partner, reflecting unequal power between partners [50] and, more broadly, their unequal status in society and the labor market [31].

Our study was conducted in the context of a long-term partnership between the World Food Programme (WFP) in Latin America and the Caribbean and the RAND Corporation, a non-profit research organization based in the U.S. This partnership was formed to conduct applied research on food insecurity and the nutritional needs of PLHIV in the region, particularly since these had been identified as barriers to ART adherence in our earlier work [53, 54]. WFP staff, RAND researchers, and local partners in the DR were involved in all phases of the research (design, data collection, analysis/interpretation, and dissemination). During formative research in four regions of the country, we found that 68% of PLHIV lived in households with moderate to severe food insecurity as measured by the validated Latin American and Caribbean Food Security Scale (ELCSA) [55, 56], and that women were disproportionately affected. In addition, assessment of dietary intake suggested low consumption of fruits and vegetables. In discussing results with local partners, the idea of an urban gardens project with WLHIV was identified as a sustainable solution. To assess the acceptability and feasibility and gain user input into such a program with WLHIV, we conducted this qualitative study in three cities in the southeastern part of the country. These cities in three contiguous provinces were chosen because their general HIV prevalence rates exceeded the national average (1.2% vs. 1.0%) [57] and due to their geographic proximity to Santo Domingo (where WFP is based) as well as to one another, which facilitated fieldwork and cost efficiencies for the study and future programming.

### Ethics statement

RAND’s Human Subjects Protection Committee and a local review board in the DR (*Consejo Nacional de Bioética en Salud del Ministerio de Salud Pública*) approved the study protocols and materials. Oral informed consent was obtained from all study participants, as advised by the RAND Human Subjects Protection Committee, since a consent document would be the only record linking the participant and the study and the principal risk would be potential harm resulting from a breach of confidentiality. Each interviewer provided a written copy of the consent statement and reviewed each section orally with potential participants and answered any questions. Oral consent was documented through a simple checklist completed by the interviewer after administering the consent process and the participant agreeing to be interviewed. Names were not included in the transcripts to protect participants’ confidentiality.

### Sampling and data collection

Between August and November 2013, we conducted in-depth, semi-structured interviews with a purposive sample of WLHIV (n = 30) who were residents of three cities in the southeast part of the country (Santo Domingo, San Pedro de Macorís, and La Romana). Average age was 38 years (range 20–56). Two trained local field workers (Dominican women with extensive experience with PLHIV and HIV programs) conducted the in-person interviews in Spanish. Interviews lasted between 60 to 90 minutes, were audio recorded (with permission), and transcribed verbatim. The criterion of data saturation was used to determine sample size [58, 59].

Eligibility criteria included age (≥18 years), being registered in one of the HIV clinics in the study, residing in an urban or semi-urban area in the three selected cities, and reporting recent food insecurity. The latter was assessed using two questions from the validated ELCSA [55, 56], namely: 1) In the last 3 months, was there a time when you were worried that you would not have enough food to eat because of a lack of money or other resources? 2) In the last 3 months, has your family been unable to eat healthy and nutritious food because of a lack of money or other resources? Women attending the clinics on the days of recruitment were referred by clinic staff to the interviewers. Interviewers explained the purpose of the study and requested permission to ask eligibility screening questions. If women met the eligibility criteria, the interviewer provided verbal and written information about the study, obtained verbal consent, and conducted the interview.

RAND researchers, WFP collaborators, and local stakeholders from PLHIV networks in the DR engaged in a collaborative process to develop the interview instrument (see S1 Table). Specifically, through in-person meetings and conference calls, a series of categories were identified as the main topics for investigation as related to information that would be useful in designing strategies to address food insecurity among WLHIV in the DR. Subsequently, the lead researchers drafted questions for each category and iterated with collaborators on wording and additional questions. Key categories included the causes of food insecurity, food and economic security, HIV treatment (including ART) and related behaviors, HIV-related stigma, intimate partner relationships, mental health, and how food insecurity affected the women.

### Data analysis

Coding procedures consisted of content analysis methods and an inductive approach [60, 61] to identify principal and emergent themes. The codebook included nine principal categories (household, neighborhood, food security status, economic security, HIV treatment and related behaviors, mental health, HIV-related stigma, reproductive health, and migration) and various sub-categories. Spanish transcripts were reviewed for accuracy by an investigator and uploaded into a qualitative data management computer software program (Dedoose). All data were analyzed in Spanish, since all team members were fluent or native Spanish-speakers. Two authors independently coded the transcripts using a codebook. A Dominican anthropologist (ST) coded all of the transcripts and the overall study’s lead investigator (KP) double coded every 3–4 interviews. Any discrepancies were resolved through consensus. Coding reports, which included the universe of tagged discourse for each category and subcategory, were reviewed by members of the analysis team to identify prominent themes. These themes were discussed among RAND and WFP team members and then with a wide variety of DR stakeholders through a formal presentation of overall preliminary results with various non-governmental and community-based organizations (including networks of PLHIV and other advocacy organizations), international assistance agencies, governmental agencies, and HIV care providers. Further analysis on the structural factors influencing food insecurity was conducted by the first two authors (KPD and DDP) and exemplary quotations that demonstrated the range of experiences were selected and translated to English for inclusion in this paper.

## Results

Food insecurity was perceived to be an intense lived experience among WLHIV in the DR. All participants spoke about the challenge of hunger and obtaining nutritious food. They also mentioned coping strategies to address food insecurity, including reducing meal portions and purchasing less costly and unhealthy food. Mothers also reported foregoing meals to provide food for their children.

A majority of the participants said the reason they experienced food insecurity was due to *a lack of economic stability* precipitated and exacerbated by social drivers including stigma and discrimination, and gender inequality. Many described labor discrimination practices, lack of gainful employment, and various types of HIV stigma that contributed to their economic instability and episodes of food insufficiency. A secondary driver of food insecurity were disruptions in social networks (family, friends, neighbors) due to HIV-related stigma, which distanced people from important social sources of food support. Below, we discuss key structural contributors to food insecurity among WLHIV: enacted and felt stigma (in the formal and informal sectors of the economy), which also translated into anticipated and internalized stigma, stigma in home and community settings, and domestic and intimate partner violence, which together undermined economic stability and access to food resources. We also discuss social and economic support described by a few women in the form of food provided by friends and family and the psychosocial benefits of HIV support groups. In Table 1, we summarize the key themes that emerged from our analysis, along with examples, coping strategies and how these precipitated or exacerbated food insecurity.

**Table 1:** Key themes, examples, coping strategies, and effects on food insecurity.

**Theme**	**Examples**	**Coping Strategies**	**Precipitates or exacerbates food insecurity through**:
***Enacted and felt stigma in formal and informal economic sectors***	Illegal testing for HIV (especially in the hotel industry and free trade zones), labor market discrimination	▪ Do not disclose HIV-positive status to employers or social network ▪ Avoid applying for jobs ▪ Quit job	▪ On-going emotional distress, fear, and worry ▪ Job loss; inability to acquire a job ▪ Economic instability
	Community gossip leading to job loss in the informal sector		
***Stigma in home and community settings***	Gossip and verbal abuse	▪ Refuse food assistance from neighbors ▪ Move away from family and community ▪ Do not disclose HIV status to close family or friends ▪ Delay or avoid healthcare treatment to avoid breaches in confidentiality or seeing/being seen by neighbors	▪ Lack of food for self and family ▪ Loss of support networks ▪ On-going emotional distress, fear, and worry ▪ Worse physical and mental health due to delays in receiving healthcare services
***Intimate relationships and domestic violence***	Abandonment	▪ Leave or lose partner; become a primary caregiver ▪ Endure abuse or emotionally unhealthy relationships to retain food and economic support ▪ Self-isolate and refuse to find a new partner	▪ Loss of financial and emotional support ▪ Physical and psychological harm ▪ Internalized stigma
	Partner violence and abuse		
**Theme**	**Examples**	**Coping Strategies**	**Addresses food insecurity through**:
***Social and economic support***	Food provided by friends and family	▪ Receive supplementary food from family and friends ▪ Receive social support from existing social networks ▪ Develop new social networks that provide information and encouragement	▪ Improved food access for self and children ▪ Improved psychological and emotional wellbeing
	HIV support groups–psychosocial support		

### Enacted and felt stigma in the formal and informal sectors of the economy: “with this condition, you know you won’t find a job…”

A key structural driver of food insecurity involved challenges in acquiring and maintaining adequate employment due to labor discrimination. Many women shared personal experiences with labor discrimination and how this influenced their food insecurity. When asked about any difficulties acquiring food, many explained that having HIV resulted in a loss of economic opportunity–“you know, with this condition, you can’t find a job”–often, regardless of education and training, as explained by this woman:

…and for the person living with HIV, it is very difficult to get a job. No matter how much of a professional you are, everything is very limited. And sometimes it is difficult to acquire food because what I earn is not sufficient…

According to respondents, labor discrimination was endemic in the tourism industry, and hotel employers were reported to engage in discriminatory practices toward PLHIV. A woman who had previously worked in a hotel said she no longer believed she could acquire a job in that setting due to her HIV status, “…I believe that if I am going to search for a job in a hotel, they won’t give it to me because I have HIV.” Several respondents reported that hotels and other service sector settings (i.e., restaurants) conducted HIV testing–or ambiguous “medical tests”–for new employees or as part of the application process. One participant described encountering this phenomenon in multiple hotels, “I applied for a job in like two or three hotels. But they would send me to take a test and then they would send me away.” Another woman indicated that she initially had success getting a job, but this changed after she was required to get tested, “I went to search for a job at a hotel, and they gave me work. But after the [medical] exam, they did not accept me.” Women perceived these tests or “medical exams” to be key barriers to gainful employment in the service sector, which contributed to their food insecurity.

Several women who were employed at the time of their diagnosis feared termination if their employer discovered their HIV status. One respondent attributed her access to food to her *lack* of disclosure at work, “My food security would have been worse if they would have found out at work and fired me. But because those at my job do not know, I have maintained my employment.” Another decided to not disclose her status to her co-workers, citing concerns about rejection, “And we always have to be hidden, or inhibited because others would reject us.” Yet another said she feared she would be fired if her employer decided to require a physical exam and discovered her HIV status, explaining that “before, I could work in any place without fear. Now, they are testing people for any little thing, and I am praying to God that they don’t test there…”

Respondents who were unemployed at the time of the interview expressed dread, embarrassment, and reluctance toward the job application process. Anticipated and internalized stigmas were prevalent, with some respondents preferring to avoid applying to jobs altogether. Participants no longer felt motivated to search for job opportunities–for example, this woman indicated that on-going food insecurity was due to her reluctance to subject herself to this obligatory test, “you don’t search for a job anymore because of it, because they will test you anywhere you go…” Others believed employers were going to reject them, disclose their HIV status to others, or treat them poorly:

…sometimes we are embarrassed to go out and search for a job because if they test us [and find we are HIV-positive], sometimes they treat us poorly, sometimes they make us become depressed. And sometimes I just prefer not to work…

Another woman, when asked about her experiences with food insecurity, responded that her decision to quit her job after her diagnosis was due to HIV-related stigma and not knowing her rights, and this resulted in food insecurity:

…when they told me, I wasn’t aware of my rights, so I left my job, I left my place of employment…and during the first few days, I received food, but after some time passed, I no longer had any money.

Enacted stigma also affected participants’ ability to acquire and maintain employment in the informal sector, where women often work. Several participants had experience as housekeepers or providing care for children in private households and described personal experiences with stigma and discrimination in household settings. However, these experiences differed from those in the formal economy since informal sector employers did not send employees to undergo physical exams or HIV testing. Instead, informal sector employers found out about their employees’ HIV status from neighbors or friends, like in the following case:

…I worked in another place and they fired me because I had HIV. I was working with a young woman who had a daughter…taking care of the daughter who was only three months old. But, as it turned out, a sister of the young woman lived near my family. Therefore, one day, the sister’s husband went to the house where I was working and saw me and asked me [what I was doing there] and I told him that I was working there and, since everyone knows [that I am HIV-positive], he told the young woman and she fired me.

### Stigma in home and community settings: “they would tell me ‘what you have to do now is die’”

Stigma and discriminatory treatment of PLHIV also manifested outside work settings, at home and in neighborhoods, and affected access to food resources. The most common form of enacted stigma consisted of gossip and verbal abuse. Several women said neighbors “talked too much” and used “bad words” against them, leading to sadness, anguish, and anger. A participant said she had previously accepted food assistance from her neighbors but was no longer accepting this kind of assistance since it often led to gossip: “There was a time when I was sick at home and people came to bring me food, but those people talked too much afterward. Therefore I no longer accept the food.” She went on to remark that although she was greatly in need of food assistance, she preferred to decline assistance from neighbors if it were accompanied with excessive gossip, “The very people who provided me with support when I have really needed it, gossip too much.”

Various types of stigma led participants to feel isolated from their previously close social networks, resulting in decreased access to social support and food resources. One woman moved away from her family after hearing disparaging remarks from a sibling, in part to keep her child from further exposure to such negativity:

If my brother was bothered with me for whatever reason, he would mention my condition and he would say, “What you should do now is die.” All of that would emotionally affect me and more so because I had a son. My son would hear that, without knowing what it was, and he would ask me why my brother had told me I should die. All of those experiences affect you deeply.

Rather than face stigma and discrimination, and risk losing access to resources for food and other basic needs, many women opted to not disclose their HIV-positive status to family or friends. Some said they had not told others due to a fear of rejection or out of embarrassment, and consequently felt “bad” about not telling their family. Others felt family members would make assumptions about their lifestyle and assume they had contracted HIV through socially unacceptable means: “Everyone thinks that if you have HIV it is because you were a whore or because your partner was a womanizer, a scoundrel.” Several women decided to not share their HIV diagnosis with others out of fear that they would be judged.

Thus, rather than receive social support from their family, several respondents felt marginalized in their own households, resulting in mental anguish and depression. The following account exemplifies such stigma and rejection:

And if I made any food at home, [my sister] wouldn’t eat it. Like I told you, she wouldn’t even carry my daughter. Nothing, I tell you, I had to go away because in truth…with that situation. And I became really thin during that time because I couldn’t…I couldn’t carry that burden.

Another participant described how her relationship with her family had deteriorated since she disclosed her HIV status:

Because when you have HIV, the family separates from you. Your family begins to treat you differently and if you have a partner, your partner also treats you poorly emotionally and in every way. Thus, it has been difficult for me to get food after I got this illness. While I didn’t have [HIV], life was, well, different.

The ramifications of verbal abuse and gossip extended beyond psychological distress and impacted aid-seeking behavior. Some participants did not like leaving their homes or would modify their schedules to avoid seeing neighbors. In one case, a respondent said she delayed seeking healthcare treatment due to fear that her neighbors would see her:

It was very difficult. I said that I would not come here [to the clinic], or to any of the health centers where I was referred. There would be a lot of people and perhaps someone from my neighborhood would see me and then I would be mocked there.

For several, the result of experiencing HIV-related stigma in household and community settings was overwhelming. Many moved to other neighborhoods after encountering enacted stigma and left behind family members and neighbors, thus disrupting their social network and potential sources of emotional and food support. Relocation could be temporary or permanent. In this case, a woman temporarily moved after her mother disclosed her HIV status to others and she was summarily shunned:

But what has affected me most was that I told my mother in secret, but my mother told a lot of people, and then they began to point me out, many people began to reject me…it began to affect me and to hurt me. I left [my province] for a while and I stayed away for about four months.

### Intimate relationships and domestic violence: “He mistreated me, there was a lot of violence and I decided to leave him.”

While spouses and partners are potential avenues for social, emotional, and economic support, including the ability to have access to stable resources for food, only two women in our sample described their partner relationships in positive terms. A participant described the value of the economic and social support provided by her spouse:

If he were like any other man, my husband would have killed me or he would have abandoned or left me. I don’t know how it came to pass, but he is by my side and he has suffered alongside me. There are times when, in terms of finances, I don’t want to give him all the burden, but he has provided for my needs to the extent that he can and in whatever he can do, he has supported me.

Instances of respectful and caring partnerships were few in our sample, and instead, a majority identified their spouses and partners as sources of stigmatization, disempowerment, and abuse. One woman said her partner abandoned her after she tested positive and he tested negative. Two others said their spouses were also HIV-positive and had passed away as a result of complications from the illness.

Several participants described intimate partner violence and abuse that led to emotional distress and separation. This violence and abuse seemed to be related both to the women’s HIV status and their vulnerability in intimate relationships. The type of abuse varied and included verbal, physical, and sexual abuse. For some, alcohol contributed to the abuse, as explained by this woman: “Oh, he was a very violent man! He drank every day. He mistreated me, there was a lot of violence and I decided to leave him.” Similarly, another woman explained how her partner’s abusive treatment was fueled by drinking and led to lasting physical marks on her body and, ultimately, their separation:

I was with a very abusive man. Yes, very abusive, and that was the reason that we separated. Because when he came out of his drunken stupor, he would get bored and he would take it out on me and I still have this mark…

Sexual abuse emerged as a key adverse event with significant negative impacts. Eight women who reported physical abuse further disclosed they had been raped by a spouse or partner. Among those who had been sexually abused by their spouse or partner, several said they did not reproach them out of fear. One participant revealed she had been sexually abused by an ex-husband who repeatedly raped her: “And when I did not want sex, to have sex, he would force me by placing his knee here above my leg, and he would force me to have intercourse.” Most of the women who had experience physical or sexual abuse said the abuse had led them to eventually leave their spouse or to no longer cohabitate with their partner due to fear or relationship dissatisfaction, as described by this woman:

Even now it is difficult [to be well nourished], ever since I left my husband because…he was negative and I was positive…and he became very violent, he was violent toward me, so I had to leave him because of the violence and now, it is difficult, since I can’t look for another man. Can you believe it, I am in this way [HIV-positive], so I can’t be in a relationship with another person.

In addition to ongoing trauma and other mental health consequences, the loss of a spouse or partner often resulted in economic difficulties and food insecurity, particularly for women with children, who then had to become the primary caregiver, as explained by this woman:

He was always the one who covered our family’s expenses. After I left him, I had to become in charge of the home responsibilities, to be both the man and the woman. It is very little what he gives me. In truth, it is not the same when a man is in the home compared to when he lives elsewhere.

One participant spoke about the advantages of having a partner strictly in terms of the economic advantages: “Yes, of course, I want a partner to help me financially, not so much for a relationship or for the sex, but I want a partner for the economic stability.”

However, many participants who expressed a desire for a life partner were hesitant to start a relationship due to their fear of infecting them with HIV. One woman spoke about her internal conflict that arose from desiring a partner, but at the same time, being fearful of infecting another person with the disease:

The only thing that has changed is that sometimes I want to have a husband, a boyfriend in my home, a husband…to have a man with me, but I can’t anymore. I can’t do it knowing that I am like this, I can’t be with a man who is healthy. Because I am not going to cause him harm…there are many women and they are lonely and they haven’t died yet.

### Social support: “if I don’t have any food, then I call my mom.”

Social and economic support along with food assistance provided by friends and family was identified as a type of resilience or buffer to combat food insecurity. A small number of participants reported having family members and friends who provided social and economic support. These women spoke with appreciation about their close social networks and the help they received from their network.

I am also very grateful to the people who have financially supported me during this time. They are still my friends. They call me if they want to give me something, and they are there for me, they have always been there for me.

Almost a third of the women said they specifically relied on their mother to provide food assistance during critical household food shortages, such as this case, “if I don’t have any [food], then I call my mom.” Another participant said her mother provided her and her children with food assistance on a regular basis:

Well, when I am at home and I don’t have a cent, I go to my mother’s and I get a plate of food and I eat. My children practically only eat with my mother because they go to school and then they go to my mother’s afterschool and eat there. My mother saves food for them every day.

Many of these same individuals, however, said that this type of assistance could be inconsistent since their mothers were also impoverished or food insecure, “Again, my mother gives me food if she has it and does not if she doesn’t have it, and I can’t really force her.” One participant shared her own experiences of having her mother-in-law provide food assistance since her own mother rejected her, she commented that her mother-in-law “is a person who always looks out for us.”

Outside of family and friends, HIV support groups—which are often linked to HIV clinics where PLHIV receive care—provided important social and emotional support. One woman attributed her improved outlook on life to participation in the support group and described being encouraged by a professional woman with HIV who also participated:

…my life began to change when I began to attend support groups, when I began to meet other people who were also living with this illness….and I would say, “That woman? She is a lawyer, she is a very pretty woman, and I would see her happy and full of self-confidence. so I would say that one day I have to feel as good as she did, and all of that helped me cope with the experience of living with HIV…

Another participant found not only emotional support from the group, but also learned of training opportunities to become a peer counselor at the clinic:

…and then I began to attend support groups and after that, my life greatly changed…Afterward, about three years later, I received many new skills, [name] invited me to many workshops at the clinic, and they sent me to volunteer with a support group. The years passed and then they employed me as a counselor where I worked until this year when the project ended.

## Discussion

We found a range of structural determinants of food insecurity among WLHIV in the DR, principally labor market discrimination in the formal and informal sectors, other manifestations of HIV-related stigma in family and community settings, and intimate partner violence. These structural factors reflect not only stigma and discrimination towards PLHIV, but also socio-cultural norms regarding women and women’s roles and gender inequalities, reflecting the intersectionality of stigma [62]. Further, our findings expand upon an earlier conceptual framework [22] by providing an in-depth view of the upstream economic and social factors that contribute to food insecurity among WLHIV in the DR. Such a perspective can help inform HIV-related policy and interventions to address food insecurity, which are particularly important given that a recent systematic review and meta-analysis found that food insecurity resulted in 29% lower odds of achieving complete HIV viral suppression [21]. Across economic contexts, food insecurity among PLHIV has also been associated with worse virologic and immunologic outcomes [20, 25], higher morbidity [63] and mortality [23, 24], and increased likelihood of depression (which has been associated with lower ART adherence and worse HIV clinical outcomes) [35, 64].

HIV-related labor discrimination in the formal and informal sectors was a commonly cited barrier to economic and food sufficiency, despite local laws that protect against such discrimination. In the formal sector, experiences with discriminatory practices were found to be rampant among women who were employed or seeking work in the tourism industry and in free trade zones (i.e., geographic areas within the country where international companies can operate with limited regulation, restriction, and taxation)—two economic hubs that employ large numbers of women [51]. Earlier studies on the tourism industry in the DR revealed a culture of silence and institutional discrimination against PLHIV among hotel managers—a quarter said they would fire employees with HIV/AIDS and some revealed (illegally) testing employees for HIV, targeting employees who handled food and were in contact with tourists [65]. Padilla referred to tourism regions in the Caribbean as “ecologies of heightened vulnerabilities” based on ethnographic research in the DR that found participation in the tourism sector can increase HIV transmission risk among low wage male workers who engage in transactional sex with male and female tourists [66, 67]. A recent qualitative study conducted in the DR with MSM (n = 16) and transgender women (n = 5) living with HIV in Santo Domingo highlighted job loss and difficulty finding employment after an HIV diagnosis due to HIV-related labor discrimination, HIV stigma, and fear of disclosure [68]. Our findings extend evidence of such discrimination to WLHIV and suggest that these practices persist in spite of national laws dating back to the early 1990s (No. 55–93) [69, 70], and most recently updated in 2011 (No. 135–11) that prohibit the use of mandatory HIV testing as part of the application process and makes it illegal to refuse to hire a person based on their HIV status. Our findings also corroborate earlier qualitative studies that found labor discrimination especially affected WLHIV and identified the need for increased enforcement of the laws aimed at protecting PLHIV [70]. For the benefit of many groups of PLHIV, there is an urgent need for multi-level programs to address illegal HIV testing and discriminatory hiring practices in the DR.

It is also important to note that even with these existing laws, PLHIV and their families may be reluctant to seek legal recourse due to a lack of disposable income to pursue legal claims as well as pervasive levels of HIV-related stigma as described by our participants. Preferred remediation strategies include legal aid services or hotlines for reporting discriminatory practices or violence [71]. In the DR, increased surveillance, reporting mechanisms, and enforcement of penalties for HIV-related labor discrimination are needed to reduce discriminatory practices. Otherwise, enacted, felt, and internalized stigma will continue to limit economic opportunities and contribute to food insecurity among PLHIV.

WLHIV in the DR are also particularly vulnerable to labor discrimination due to their over-representation in jobs in the informal sector, which often lack a formal contract and are often not under the purview of labor regulations and laws [51, 69], making it more difficult for victims to pursue legal recourse. Informal jobs may also present additional health risks that leave PLHIV more vulnerable in the long run [22]. Further, these structural factors exacerbate women’s vulnerabilities and may lead them to engage in transactional sex, exposing them to risks such as sexual abuse and infectious disease [12, 34]. Another qualitative study suggested that women often resort to sex work to address household and individual food insecurity, resulting a cycle of food insecurity, HIV risk, and social marginalization [72].

Among our participants, stigma and discrimination also resulted in social network disruptions, internalized stigma, and poor mental health. Previous quantitative work among WLHIV in the DR has found that internalized stigma is strongly and positively associated with depression, perceived community stigma and perceived family stigma [73]. The consequences of rejection from family, friends, and neighbors include sadness, perceived lack of value, and disempowerment within an interpersonal network [69] contributing to their fear of disclosure [31]. Further, internalized stigma resulted in food insecurity by leading to social withdrawal from family and community support networks that could be instrumental in increasing access to food, as has been suggested from quantitative research [40]. Given these consequences to disclosure, it is thus not surprising that some respondents decided to keep their HIV status hidden from their communities and families, similar to what has been found in other studies [74, 75]. Among those who did disclose their HIV status, partner abandonment or separation also deprived women of an important emotional and financial support, which further contributed to their food insecurity. Thus, women must often cope not only with HIV and food insecurity, but also with the loss of intimate relationships, friends, and family, which can be devastating. WLHIV are often compelled to remain in abusive relationships precisely because they rely on men for food for themselves and their children, as noted by other qualitative studies [76].

On the other hand, support from partners and family members, though less frequent among our participants, proved to be key sources of emotional and financial support for these women, as well as from support groups sponsored by clinics and the networks of PLHIV. Support groups have previously been identified as important strategies to decrease isolation and feelings of shame and to expand social networks with positive emotional and physical health outcomes for WLHIV [77]. Future research is needed to identify ways to expand such support through clinic-based and community-based interventions.

The study has some limitations. As is typical with qualitative studies, the findings reflect the experiences of the sample and may not be generalizable to the population of WLHIV who receive services from the HIV clinics where recruitment occurred or in the country. Further, since we elected to sample women from the clinics where they received care, our study may have not adequately captured the experiences of WLHIV who are out of care or are less adherent to care (e.g., attend the clinic infrequently or have abandoned treatment), who may be even more vulnerable to food insecurity and poor HIV outcomes. We also focused on women in urban and peri-urban areas, which may limit the types of industries encountered and thus structural discrimination experiences.

### Policy and program implications

The findings add to the growing body of literature in support of developing interventions to address structural barriers among PLHIV in the DR [67, 68]. Strong national policies addressing food insecurity and nutritional needs among PLHIV are still lacking in the DR and many other countries, despite recognition that good nutrition and food security are crucial for HIV outcomes and adherence to antiretroviral therapy [78]. Most programs aimed at addressing food insecurity among PLHIV in low-resource settings have involved providing food supplementation, with results generally showing benefits for both nutritional well-being and ART adherence [79]; however, such efforts are typically not sustainable in the long term and do not address the underlying structural factors associated with food insecurity. Income-generating interventions may provide more sustainable approaches to addressing food insecurity [38]. However, such interventions will likely be most effective if they also help address some of the social drivers of food insecurity–for example, the stigma and discrimination towards WLHIV in the labor market and in their families and communities–in addition to economic drivers.

In addition, addressing broader gender inequality issues are needed to improve economic and social conditions for WLHIV. In the DR, this means developing new employment opportunities and increasing women’s access to sectors beyond the tourism industry and informal sectors (e.g., public works) [51]. Such reduction of economic and social inequalities will benefit WLHIV as well as the health and wellbeing of marginalized populations and individuals in general [31, 37]. Further, cross-sectoral efforts are important to truly address the links between gender-based violence and HIV. For example, integration of health and social care services and standardized screening and referrals procedures would enable providers to respond appropriately; legal reform (e.g., making marital rape a criminal offense) and training and disciplinary procedures to address police inaction and inappropriate statements and behaviors towards women who seek assistance for gender-based violence is also needed [80, 81].

There is also a need for greater awareness and education around the rights of women and existing discrimination laws to protect the rights of PLHIV, as well as research to quantify the extent of the problem. Enforcing current laws and penalizing discrimination by employers can help reduce discriminatory practices. Further, reports of illegal testing indicate that health professionals or at least diagnostic laboratories are also complicit, since testing is being done without any pre- or post-counseling. Research and routine surveillance and reporting practices to document and track labor discrimination practices against vulnerable populations, such as PLHIV, could help inform needed areas of intervention. Population-based studies at the national level that assess attitudes and behaviors toward PLHIV as well as the perceived experiences of PLHIV can also lead to a stronger understanding of the problem of HIV-related stigma [31] and related consequences and inform country-specific campaigns and strategies.

## Conclusion

WLHIV in the DR face many structural barriers to achieve food security for themselves and their families, including but not limited to economic barriers, requiring multi-level action to protect their health and human rights. In the short term, social protection strategies are needed that promote food security and economic stability among PLHIV, particularly women, as a buffer against both societal and interpersonal discrimination. Longer term strategies must address stigmatizing attitudes and discriminatory practices towards PLHIV and in particular towards WLHIV within the formal and informal labor sectors and the broader community, as well gender-based violence towards WLHIV that reflects the intersectionality of stigma. Long-lasting change will also likely require reduction of social and economic inequalities at the national and international levels.

## Supporting information

S1 Table Project on food and nutritional security of women in the Dominican Republic: Interview guide (English translation). (PDF)
