# Supplementary material for: Factors contributing to food insecurity among women living with HIV in the Dominican Republic: A qualitative study
Source: PLoS One. 2017 Jul 25;12(7):e0181568. doi: 10.1371/journal.pone.0181568 (PMC5526502; doi:10.1371/journal.pone.0181568)
Supplement: S1 Table — (PDF) [file pone.0181568.s001.pdf]

Project on Food and Nutritional Security of Women in the Dominican Republic
Interview guide
(Translated from Spanish to English)

Housing and neighborhood

First, I would like you to tell me about your housing and neighborhood

1. Tell me where you live now and where you lived before.
   - Could you describe your home?
   - Who is currently living with you at your home, including adults and children?
   - Are you the owner of the home in which you live or rent?
   - Could you describe the different places you lived before this house?
   - Could you describe for me if you migrated from a rural area? How long ago? Why did your move to the urban area?
   - Do you have space to grow your own food?

2. Could you describe your neighborhood or area where you live?
   - How is your neighborhood in terms of its physical characteristics?
   - Who lives in this neighborhood?
   - Do you have access to the institutions, businesses and services you need in your area?

Food Security

Let us now turn to the food and nutritional situation of your family. Tell me more about your food situation: where you get the foods you eat and what problems you have getting the quantity or type of food you want.

3. What difficulties or challenges have you had in getting enough food?
   - [IF SPEAKS ABOUT DIFFICULTIES] Why do you think you have had these difficulties?
   - [IF DOES NOT SPEAK ABOUT DIFICULTIES] Why do you think you have not had difficulties?

4. What difficulties have you had in obtaining a high quality diet and variety (diverse foods)?
   - [IF SPEAKS ABOUT DIFFICULTIES] Why do you think you have had these difficulties?
   - [IF DOES NOT SPEAK ABOUT DIFFICULTIES] Why do you think you haven't had any difficulties?

5. Do you receive help from any assistance program from the government, institution, or other close people who provide you with food?
   - [IF RECEIVES HELP] Can you describe to me what kind of help you get from these sources? How do they contribute to the food situation of your home?
   - [IF DOES NOT RECEIVE HELP] What are the reasons why you do not receive help? For example, it is that you do not need any help, prefer not to receive help even if you need it, you are not eligible, or do not know where to get help, etc.

6. In what ways do difficulties in getting food of sufficient quantity and quality affect your health, both physical and psychological?
   - Can you tell me about a time when lack of food affected your health?
   - [IF THERE WERE NO DIFFICULTIES] In what ways do you think your diet and nutrition affect your health? Were there times in the past that you did not feel good for lack of good nutrition? Can you tell me about this time?

7. Can you describe how the food is distributed among the members of your family?
   - In other words, how do you decide on the amount, when and what foods each person has to eat? (E.g., the participant, her partner, her sons, her daughters, the family of the couple or relatives).
   - Some women put the needs and food of their children or their partner before their own needs. Have you had this experience when foods have been scarce?
     - [IF ANSWER IS AFFIRMATIVE] Can you describe how the food is distributed in this situation?

8. Did you experience changes or difficulties in obtaining adequate food and nutrition since you learned that you were HIV positive?
   - Can you describe these changes or difficulties?
   - What do you attribute to these changes to?

9. Have you had any migration experiences?
   - [IF AFFIRMATIVE] How has this experience affected your eating and good nutrition?

10. Tell me about situations in which you have grown the food you were going to eat.
    - Could you describe any experiences you have had with agriculture, family or community gardens, or the rearing of chickens or other animals
    - (IF THE ANSWER IS AFFIRMATIVE) Which foods do you grow? Where did you grow them?
      - Did you have help from an institution, family or friend to grow food? What help did they provide: seeds, soil preparation, tools, etc.
    - (IF THE RESPONSE IS NEGATIVE) If you had the opportunity to grow food, would you be interested in participating? What foods would you like to grow? Who would you like to participate? What do you understand that you would need to start: space, tools, seeds, etc.?

Financial Situation

11. I would like to hear more about your financial situation. Tell me more about your current financial situation, including how you obtain money to meet your needs. Your responses are entirely confidential.
    - Can you tell me how you distribute your income so that you can buy the basic necessities of your family?
    - What basic needs have your family had to sacrifice for not having enough income?
    - How has your work situation changed since knowing your HIV status?
    - Has your economic situation changed because of your living with HIV?
    - Could you tell me how the income is distributed within the family?

- For example, if you work, who do you share with or do you deliver money to? What if your partner or other family member works?
- Do you or your family own land or property?
    - [IF YES] Could you describe these lands or properties? Where are they located? Who has responsibility for them and who receives the benefits of them?
- Have there been disagreements about your land and property?

Adherence to Treatment and Care

12. Now we are going to transition to discuss issues around the treatment of your HIV. I want to start by taking about your experiences taking antiretroviral drugs (ARVs). Could you tell me about this? **[IF PARTICIPANT INDICATES THEY ARE NOT TAKING ARVs, SKIP THIS QUESTION]**

    - What difficulties have you had in taking your ARVs regularly?
        - Could you tell me about once time that you had difficulty taking your medications regularly? What was the problem? How did you solve it?
        - [IF DOES NOT REPORT DIFFICULTIES] Why do you think you have never had difficulties taking your medications regularly?

    - Was there ever a time that you did not take your doses at the indicated time or did you stop taking your doses temporarily?
        - [IF ANSWER IS AFFIRMATIVE] What was the reason? Do you have problems taking your doses now?

13. Now I want to hear about your experiences going to the clinic. Could you tell me about this?
    - What difficulties have you had in attending medical visits or coming to the clinic?
    - Are there are times that you do not go to the appointment? What are the reasons why this happens?

Mental Health, HIV-Related Stigma and Social Support

(Thank you). Now I want us to talk about how you feel day to day.

14. Could you tell me how you feel, how is your mood? [ASK ABOUT SPECIFIC SITUATIONS]
    - What things in your life affect how you feel day to day?
    - Have you felt down, sad or depressed recently? Why? Could you describe what situations make you feel that way?
    - Have you felt confused, distressed, or stressed recently? Why? Could you describe what situations make you feel that way?
    - Have you felt hope, joy or peace recently? Why? Could you describe what situations make you feel that way?
    - Have you felt you have the ability to adapt to difficult situations?
    - How does your food or nutritional situation affect how you feel mentally or emotionally?

Thank you for sharing your health information with me. Now I would like to talk about your experiences with HIV.

15. How has your experience of living with HIV been? [ASK ABOUT SPECIFIC SITUATIONS]
    - Could you tell me when you were told about your HIV diagnosis? How did you deal with your diagnosis in the beginning? How has your experience changed over time?
    - Could you tell me who you have told about your HIV diagnosis?
        - Why did you tell these people?
        - How did they react?
        - [IF HAS NOT TOLD ANYONE] Why? What do you think would happen if you told someone about your HIV status?
    - How are your relationships with family, friends, and community members since you learned of your positive HIV status?
    - Have you had feelings of guilt or embarrassment about your HIV-positive condition?
        - [IF ANSWER IS AFFIRMATIVE] Could you tell me more about these feelings? Why do you think that you feel this way?
    - Have you been a victim of verbal abuse or physical violence because of your HIV positive status?
        - Could you tell me about this?

Sexual health and relationship power

Next, I would like to hear about decision-making in your family and your intimate sexual relationships. Please remember this is entirely confidential and we are used to hearing about these topics, so don’t worry about being shy or being judged.

16. First, I'd like to understand more about your partner or intimate relationships. Could you describe your current relationship?
    - Do you have a formal partner? Could you tell me a little about your partner?
    - How satisfied are you with your relationship? Why?
    - Do you also have some other partners?
        - [IF ANSWER IS AFFIRMATIVE] Could you describe me a little more about these relationships?

    - Have you had other partners in the past?
        - [IF ANSWER IS AFFIRMATIVE] Could you describe me a little more about these relationships?

17. Now let's talk about how decisions are made in your relationship or in your home. How are decisions usually made within the couple relationship, or at home? [IF DOES NOT HAVE A PARTNER, ASK ABOUT DECISION MAKING WITH OTHER HOUSEHOLD MEMBERS, FOR EXAMPLE, WITH HER MOTHER]
    - What kinds of decisions do you make for yourself and what kinds of decisions do other people make (your partner, or other family members)?
    - For example, how decisions are made about work, how to spend money, what to do with your time, possessions, or children.

18. Now I'm going to ask you about your intimate experiences with your partner. Could you tell me about your experiences in making decisions related to sexual relations with your partner (or partners)?
    - How do you decide when to have sex? Who decides? How do you feel about this situation?
    - How do you decide what to do during sex, including condom use? Who decides? How do you feel about this situation?

19. Other women have told us about being afraid of refusing to have sex with their partner. Can you talk about your own experiences with situations like this, recently or in the past? [ASK ABOUT SPECIFIC SITUATIONS]
    - What barriers prevented you from saying no?
    - How are these experiences related to food insecurity or economic instability?

20. Women are sometimes ill-treated or victims of violence in their relationships. Have you ever experienced this situation in a relationship? [TAKE NOTE IF THE PARTICIPANT SAYS THAT SHE HAS BEEN A VICTIM OF ABUSE AND REFER HER TO SERVICES AT THE END OF THE INTERVIEW]
    SURVEY:
    - [IF ANSWER IS AFFIRMATIVE] I am very sorry that he suffered such abuse. Could you tell me a bit more about your experience? How did you deal with this situation?

21. Some people remain in a relationship where they have been ill-treated while others decide to leave. Can you talk about your own experiences? [TAKE NOTE IF THE PARTICIPANT SAYS THAT SHE HAS BEEN A VICTIM OF ABUSE AND REFER HER TO SERVICES AT THE END OF THE INTERVIEW]

    - [IF SHE LEFT THE RELATIONSHIP] Could you tell me more about your decision to leave this difficult relationship and how did you make this decision?
    - [IF SHE DID NOT LEAVE THE RELATIONSHIP] Could you tell me more about your decision to stay in this relationship and how did you make this decision?
    - How do these experiences and decisions relate to food insecurity or economic instability?

That information helps us a lot. Thank you.

We are getting to the end of the interview. Is there any other topic that you would like to talk about?

Conclusion

- Is there something that you started to say about any topic that you did not get to elaborate on?
- May I review the interview guide quickly and make sure we have spoken about everything?
- Thank you very much for your time. Do you have any questions about this interview before we end today?
